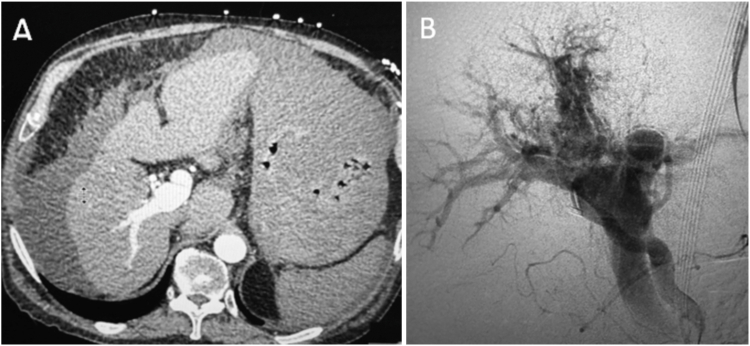# Hematemesis as a Result of Hepatic Arterioportal Fistula Formation

**DOI:** 10.1016/j.gastha.2024.10.019

**Published:** 2024-10-22

**Authors:** Hannah Zuercher, Juan Gonzalez, Andreas Zori

**Affiliations:** 1 Divison of Internal Medicine, University of Florida College of Medicine, Gainesville, Florida; 2 Division of Gastroenterology, Hepatology, and Nutrition, University of Florida College of Medicine, Gainesville, Florida

A 77-year-old man with history of cirrhosis due to alcohol and hepatitis C presented with hematemesis and melena, requiring vasopressors and blood transfusions. Computed tomography scan of the abdomen/pelvis described no active gastrointestinal hemorrhage, an 8.6-cm heterogeneous hepatic mass concerning for malignancy, and a right portal vein filling defect, felt to represent bland versus tumor thrombus (Figure A). Esophagogastroduodenoscopy demonstrated significant bleeding in the gastric fundus and type 2 gastroesophageal varices oozing/spurting blood, no endoscopic intervention was performed. Sengstaken–Blakemore tube was placed for stabilization. Upon further imaging review with interventional radiology, suspicion was raised for an arterioportal fistula. Angiogram demonstrated a large hepatic arterioportal fistula (Figure B). Partial embolization was performed with significant flow reduction. However, complete embolization was not possible due to the size of the shunt and involvement of the entire hepatic artery due to supply from right/middle/left hepatic artery branches. Given the patient’s poor prognosis, comfort care was chosen.

This case highlights the importance of considering arterioportal fistula formation as a cause of portal hypertension leading to variceal bleeding in patients with hepatocellular carcinoma. Although transcatheter arterial embolization is a potential treatment for arterioportal fistula, it may not be feasible to embolize all involved areas.

**Figure:**